# 

**Published:** 1898-03

**Authors:** 


MARCH, 1898.

*Vol. XVI.* *No.* **59.**

LIBRARY OF CONGRESS
185036
APR 25 1898
SMITHSONIAN DEPOSIT.

### THE BRISTOL MEDICO-CHIRURGICAL JOURNAL

**A Quarterly Journal of the Medical Sciences for the West of England and South Wales**

*PUBLISHED UNDER THE AUSPICES OF*
*THE BRISTOL MEDICO-CHIRURGICAL SOCIETY.*

"Scire est nescire, nisi id me

Scire alius sciret."

*Editor:* R. SHINGLETON SMITH, M.D., B.Sc.
WITH WHOM ARE ASSOCIATED
J. MICHELL CLARKE, M.A., M.D., F. R. CROSS, M.B.,
W. H. HARSANT, AND JAMES SWAIN, M.S., M.D.
*Assistant-Editor:* L. M. GRIFFITHS.
*Editorial Secretary:* B. M. H. ROGERS, B.A., M.D., B.CH.

BRISTOL: J. W. ARROWSMITH
LONDON: J. & A. CHURCHILL, 7 GREAT MARLBOROUGH STREET.

*Price One Shilling and Sixpence.*

